# MASEP gamma knife radiosurgery for secretory pituitary adenomas: experience in 347 consecutive cases

**DOI:** 10.1186/1756-9966-28-36

**Published:** 2009-03-11

**Authors:** Heng Wan, Ohye Chihiro, Shubin Yuan

**Affiliations:** 1 Department of Neurology and Functional neurosurgery, West China Fourth hospital, Sichuan University, Chengdu, 610041, PR China; 2 Functional and Gamma Knife Surgery Center, Hidaka Hospital, Takasaki, Gunma, Japan; 3 Department of Stereotactic and Functional Neurosurgery, Chengdu Air-force 452 Hospital, Chengdu, 610010, PR China

## Abstract

**Background:**

Secretory pituitary adenomas are very common brain tumors. Historically, the treatment armamentarium for secretory pituitary adenomas included neurosurgery, medical management, and fractionated radiotherapy. In recent years, MASEP gamma knife radiosurgery (MASEP GKRS) has emerged as an important treatment modality in the management of secretory pituitary adenomas. The goal of this research is to define accurately the efficacy, safety, complications, and role of MASEP GKRS for treatment of secretory pituitary adenomas.

**Methods:**

Between 1997 and 2007 a total of 347 patients with secretory pituitary adenomas treated with MASEP GKRS and with at least 60 months of follow-up data were identified. In 47 of these patients some form of prior treatment such as transsphenoidal resection, or craniotomy and resection had been conducted. The others were deemed ineligible for microsurgery because of body health or private choice, and MASEP GKRS served as the primary treatment modality. Endocrinological, ophthalmological, and neuroradiological responses were evaluated.

**Results:**

MASEP GKRS was tolerated well in these patients under the follow-up period ranged from 60 to 90 months; acute radioreaction was rare and 17 patients had transient headaches with no clinical significance. Late radioreaction was noted in 1 patient and consisted of consistent headache. Of the 68 patients with adrenocorticotropic hormone-secreting(ACTH) adenomas, 89.7% showed tumor volume decrease or remain unchanged and 27.9% experienced normalization of hormone level. Of the 176 patients with prolactinomas, 23.3% had normalization of hormone level and 90.3% showed tumor volume decrease or remain unchanged. Of the 103 patients with growth hormone-secreting(GH) adenomas, 95.1% experienced tumor volume decrease or remain unchanged and 36.9% showed normalization of hormone level.

**Conclusion:**

MASEP GKRS is safe and effective in treating secretory pituitary adenomas. None of the patients in our study experienced injury to the optic apparatus or had other neuropathies related with gamma knife. MASEP GKRS may serve as a primary treatment method in some or as a salvage treatment in the others. However, treatment must be tailored to meet the patient's symptoms, tumor location, tumor morphometry, and overall health. Longer follow-up is required for a more complete assessment of late radioreaction and treatment efficacy.

## Background

Pituitary adenomas are common lesions and represent 20% of all primary brain tumors[1,2]. The epidemiological studies have demonstrated that nearly 20% of the general population harbor pituitary adenomas[3,4]. Pituitary adenomas are broadly classified into two groups[5]. In the first category are those that secrete excess amounts of normal pituitary hormones and present with a variety of clinical syndromes depending on the types of hormones secreted. Meanwhile, some macroadenoma may present with pressure symptoms, often increase in size if untreated, and in some rare cases they may cause symptoms related to mass effect in which the optic nerves and chiasm are compressed[6,7]. The second category of pituitary adenomas is nonfunctioning adenomas that do not secrete any known biologically active pituitary hormones. Patients can also suffer hypopituitarism secondary to compression of the normal functioning pituitary gland[8].

In the treatment of pituitary adenomas the goal is to remove the tumor mass or arrest further growth and when present normalize hormonal hypersecretion. Transsphenoidal surgery is established as one of the most reliable treatment modalities. This modern microsurgical technique can reduce tumor mass to protect surrounding structures from potential compression, and achieve the endocrinological cure of the symptoms caused by hormone secreting tumors. Long term tumor control rates after transsphenoidal excision alone vary from 50 to 80%[9]. However, in some cases, many patients are already in poor physical condition caused by extended production of the excess pituitary hormones, and general anesthesia itself sometimes brings a certain risk for them. Also, they often show invasion to surrounding structures including cavernous sinus. And for these types of pituitary adenomas, incomplete tumor resection or recurrence as a result of tumor invasion into surrounding structures is quite common[10].

In recent years, gamma knife radiosurgery(GKRS) has emerged as an important treatment modality in the management of secretory pituitary adenomas with its high efficacy. Radiosurgical treatment may deliver a high dose to the adenomas with high accuracy and may not influence the nearby neural structures to induce neurological defect[11]. Recently, more and more reports have detailed treatment results for secretory pituitary adenomas with GKRS, and there have been a number of reports of GKRS as a primary treatment for secretory pituitary adenomas[12]. However, most results of these reports were based on the gamma knife with radioactive source statically, and the time of the follow-up varied from months to years. This article reviews our 10 years of clinical experience in performing rotary gamma knife in patients with secretory pituitary adenomas. The focus of this research is to define accurately the efficacy, safety, complications, and role of rotary gamma knife for treatment of secretory pituitary adenomas.

## Methods

### Characteristic of the patients

Between 1997 and 2007, 1681 patients with a diagnosis of secretory pituitary adenoma were treated with MASEP rotary gamma knife(MASEP instruments, Inc., Shenzhen, P.R. China) in our medical center. The patients with secretory pituitary adenoma treated in our studies are those loss convenience, intolerant of or resistant to medical therapies. Some of them were evaluated ineligible for neurosurgery because of body health and the others rejected to surgery on private choice or economic condition. 347 patients under medical therapies irregularly less than 3 months after MASEP GKRS and getting follow-up with at least 60 months were taken in our study, and those with follow-up less than 60 months or taken medical therapies regularly after MASEP GKRS were excluded. Our study population comprised 162 men (46.7%) and 185 women (53.3%). Their age ranged from 17 to 86 years (mean 41.8). The patients presented with a 1- to 19-year history (mean 2.7). In 47 of these patients some form of prior treatment such as transsphenoidal resection, or craniotomy and resection had been conducted. The others were deemed ineligible for microsurgery because of body health or private choice, and MASEP GKRS served as the primary treatment modality. Endocrinological, ophthalmological, and neuroradiological exams were taken for all of them. The diagnosis was made on the basis of magnetic resonance imaging (MRI) findings, endocrinological exam findings, pathological findings (available for postoperative patients), and their clinic history. Of these patients treated, 68(19.6%) had a diagnosis of adrenocorticotropic hormone-secreting adenomas, 176(50.7%) had a diagnosis of prolactinomas, and 103(29.7%) had growth hormone-secreting adenomas. The mean follow-up period was 67.3 months (range 60~90 months) (Table 1).

**Table 1:** Characteristics of patients with pituitary adenomas treated with MASEP GKRS

Characteristic	Value(%)
The statistics of the population	
Sex	
male	162(46.7)
female	185(53.3)
Mean age(yrs)	41.8 (range17~86)
Mean history(yrs)	2.7(range1 to 19)
No. of previous treatments	
transsphenoidal resection	27*
craniotomy and resection	23
Mean follow-up after GKRS(mos)	67.3 (range 60~90)
Type of adenomas	
ACTH adenomas	68(19.6)
microadenoma(size, cm^3^)	21 (0.8~1.1)
macroadenoma(size, cm^3^)	47 (1.2~6.4)
Prolactinomas	176(50.7)
microadenoma(size, cm^3^)	0
macroadenoma(size, cm^3^)	176(1.2~17.9)
GH adenomas	103(29.7)
microadenoma(size, cm^3^)	0
macroadenoma(size, cm^3^)	103(2.3~21.5)

*three patient had performed repeated craniotomy and resection

### Gamma knife processing

Patients were fixed in the Leksell stereotactic head frame under administration of local anesthesia. Before treatment, a high-resolution MRI with gadolinium-enhancement to obtain precise information on the shape, volume, and the three-dimensional coordinates of the tumors and the surrounding anatomic structures is performed. Radiosurgery was performed using the MASEP rotary gamma knife. MASEP rotary gamma ray stereotactic extracranial system is equipped with 25 Co-60 sources. Each source is formed by certain amount of Φ1 × 1 cobalt granules welded into 2 layer stainless steel casing through argon fluorine welding technique to make it seal-tight. The total combined initial loading activity is 240.5 TBq ± 10% (6500 Ci ± 10%). Source specific activity is 300 Ci/g. Source active zone is Φ3.1 × 30. At initial loading the water-absorption dose rate at focusing point is greater than 3 Gy/min. 25 cobalt sources are placed in the collimator passages. The commercially available software, MASEP Gamma-Plan (MASEP instruments, Inc., Shenzhen, P.R. China) was used for complex dose planning. The radiosurgical planning was done jointly by neurosurgeons and radiation oncologists. Dose planning requires delineation of the targets and the adjacent structures, especially the optic chiasm. Though the MASEP gamma knife has five collimator sizes, 4, 8, 14, 18 and 22 mm, the 4 mm and 8 mm collimator were used commonly. The day before MASEP GKRS, patients were claimed to take 1.5 mg hexadecadrol. The day after MASEP GKRS, patients were desired to take intervenous drop infusion of 250 ml mannitol plus 10 mg hexadecadrol (twice a day) for 3 days to avoid radioreaction. Then they were discharged and could return to their daily lives without any neurological deterioration.

### Treatment planning

Tumor volume was 0.8~21.5 cm^3^(mean 5.2 cm^3^). For the purpose of both growth control and hormonal remission, secretory pituitary adenomas were usually irradiated more than 12 Gy (range 12~35 Gy) at the tumor margin. The whole tumor was covered within 50~70% isodose lines. The dosimetric goal in every case was complete tumor coverage. The prescribed marginal dose had to be decreased occasionally to keep the dose less than 10 Gy to the optic nerve, chiasma, and tract to avoid radiation-induced visual disturbances, less than 12 Gy to the brainstem and less than 25 Gy to the internal carotid artery (Table 2).

**Table 2:** MASEP GKRS plan for patients with pituitary adenomas(mean)

Type	Cases	Margin dose(Gy)	Treatment isodose(%)	Tumor coverage(%)
ACTH	68			
microadenoma	21	15~28(18.9)	50	100
macroadenoma	47	18~35(24.9)	50~70(54.7)	70~100(95.3)
PRL	176			
microadenoma	0	0	0	0
macroadenoma	176	15~35 (22.4)	50~70(55.3)	64~100(93.3)
GH	103			
microadenoma	0	0	0	0
macroadenoma	103	12~30 (21.4)	50~70(57.6)	55~100(88.6)

### Clinical observation

After the treatment of MASEP GKRS, follow-up was scheduled at intervals of 6 months, 1 year and annually thereafter. The visit routinely assessed the change in symptoms after treatment, endocrinological examination and neuroradiological study.

In our series to evaluate ACTH-producing pituitary adenomas, we utilized the 24 h urine cortisol collection not excess of 200 μg/dL (550 nmol/dL) and the plasma cortisol level less than 2.5 μg/dL (69 nmol/dL) as the criteria for endocrinological evaluation. For patients treated with prolactinomas, we used normal serum prolactin level for gender as cure criteria and the normal PRL range for nonpregnant women is <500 mU/L (20 μg/L) and for men <300 mU/L (12 μg/L). Meanwhile, we used the guidelines for a remission or cure as the GH level less than 1 ng/ml(2.5 mU/L) after glucose ingestion and a normal serum type-1 insulin like growth factor(IGF-1) when matched for age and gender to define the results of radiosurgery for patients with acromegaly.

After irradiation of pituitary tissue, regular surveillance is needed to detect development of hypopituitarism, particularly GH deficiency. Basal pituitary profiles, including measurement of TSH, ACTH, gonadotropins, growth hormone, IGF-1 and assessment for the clinical features of GH deficiency or consequent gonadal failure, were performed regularly on follow-up.

### The statistical analysis

Statistical analysis was performed with the aid of commercially available software (StatView 4.5.1; Abacus Concepts, Inc., Berkeley, CA).

## Results

**Figure 1:**
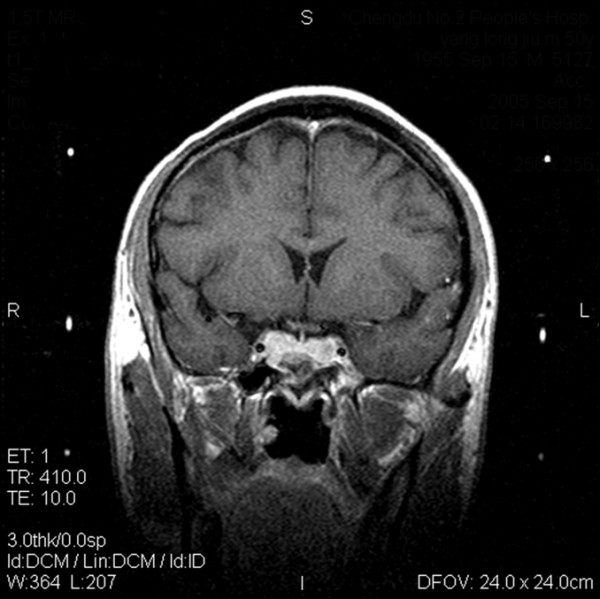
**Typical MRI scan changes in ACTH adenoma**. Coronal T1-weighted postcontrast MRI scan at left and right, obtained in Patient 1, a 30-year-old man who presented with ACTH adenomas and consistent headache 2 years before undergoing GKRS. An enhancing mass lesion is seen in the sella turcia with extension to bilateral internal carotid artery. Patient 1's serum ACTH level was 381.6 pg/ml, and his blood pressure was over 180/120 mmHg. The patient was treated with MASEP GKRS, and MRI was performed for treatment planning. 26 Gy defined to the 50% isodose line is used to cover the full extent of the pituitary tumor in all three planes.

**Figure 2:**
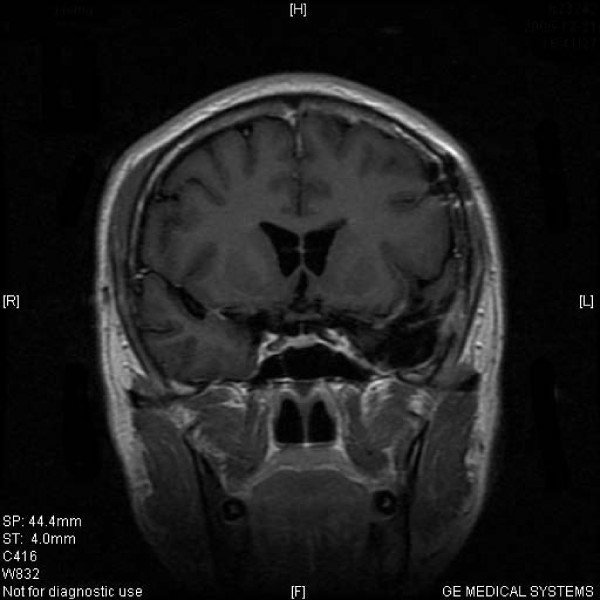
**Typical MRI scan changes in ACTH adenoma**. No enhancing mass lesion is seen in the sella turcia under the T1-weighted postcontrast MRI scan performed 2 years after GKRS. Patient 1's clinical symptom did improve. His serum ACTH level came down to 40.4 pg/ml, and his blood pressure was controlled within 140/80 mmHg.

**Figure 3:**
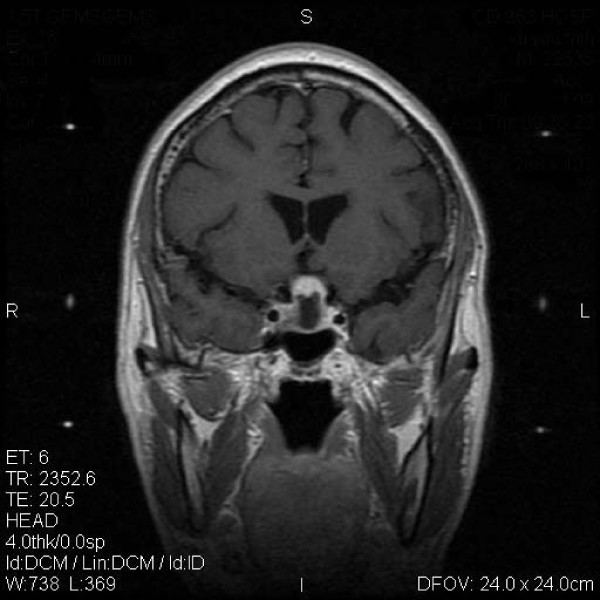
**Typical MRI scan changes in prolactinomas adenoma**. Coronal T1-weighted postcontrast MRI scan at left and right, obtained in Patient 2, a 27-year-old woman who presented with prolactinomas adenomas and amenorrhea-galactorrhea 4 years before undergoing MASEP GKRS. An asymmetrically enhancing mass lesion is seen in the sella turcia with extension to bilateral internal carotid artery. Patient 2's serum prolactin level was 183.7 ng/ml. The patient was treated with MASEP GKRS twice because of the huge volume of the mass. The second MASEP GKRS was performed 1 year after the first one. The tumor was treated separately with the lower and upper part in order to protect the optic chiasma. MRI was performed for treatment planning. 25 Gy defined to the 50% isodose line is used to cover the lower part of the pituitary tumor in the first treatment, and 18 Gy defined to the 50% isodose line is used to cover the upper part of the pituitary tumor in the second time.

**Figure 4:**
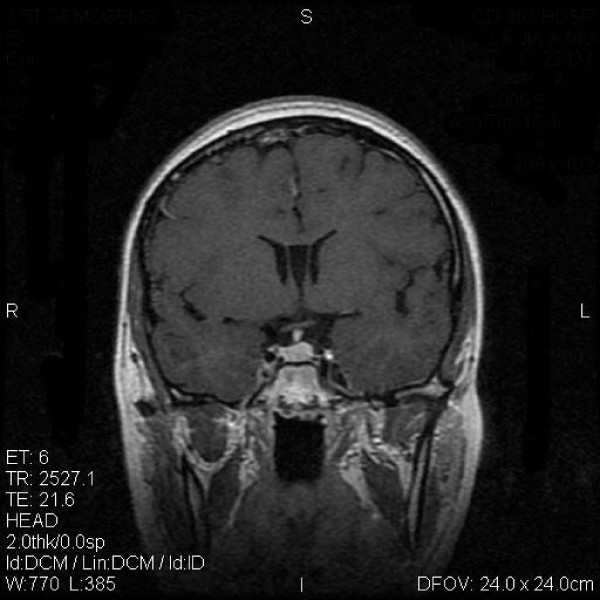
**Typical MRI scan changes in prolactinomas adenoma**. An enhancing mass lesion is seen in the sella turcia under the T1-weighted postcontrast MRI scan performed 1 year after MASEP GKRS, but the volume of the mass had collapsed for more than 50%. Patient 2's clinical symptom did improve. Her serum prolactin level came down to 14.5 ng/ml, and she got gestation and delivered a healthy baby recently.

**Figure 5:**
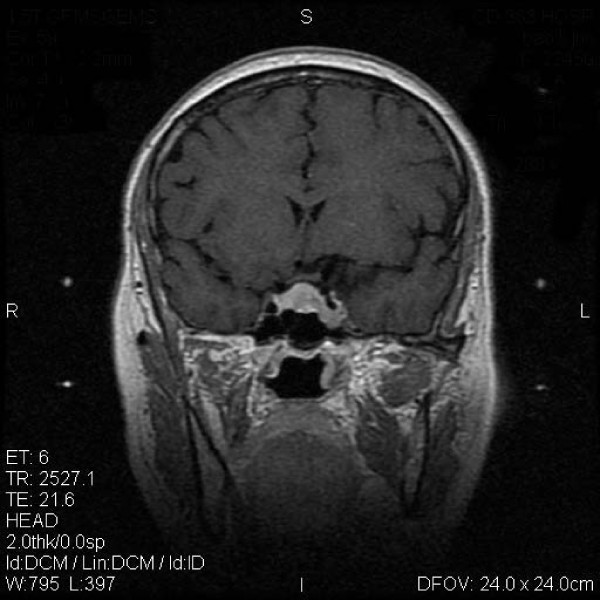
**Typical MRI scan changes in GH adenoma**. Coronal T1-weighted postcontrast MRI scan at upper left and right, obtained in Patient 3, a 33-year-old man who presented with GH adenomas and acromegaly 7 years before undergoing MASEP GKRS. (Figure 5) An enhancing mass lesion is seen in the sella turcia with extension into the left cavernous sinus. Patient 3's serum growth hormone level was 497.3 ng/ml initially. He was treated with transsphenoidal surgery, and the tumor relapsed shortly with the serum growth hormone level reduced to 130.2 ng/ml. The patient was treated with MASEP GKRS, and MRI was performed for treatment planning. 20 Gy defined to the 50% isodose line is used to cover the full extent of the pituitary tumor in the first radiosurgery, and 28 Gy defined to the 50% isodose line is used to cover the pituitary tumor in the second time one year later.

**Figure 6:**
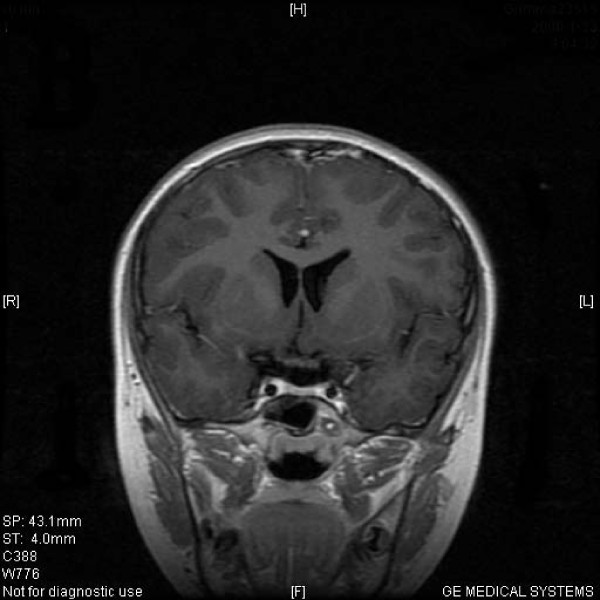
**Typical MRI scan changes in GH adenoma**. No significantly enhancing mass lesion is seen in the sella turcia under the T1-weighted postcontrast MRI scan performed 1 year after the second MASEP GKRS. Patient 3's clinical symptom did improve. His serum growth hormone level was lower than 10 ng/ml.

MASEP GKRS was tolerated well in these patients. Acute radioreaction was rare and 17 patients had transient headaches with no clinical significance. Consistent headache was noted in 1 patient 4 years after radiosurgery and persisted for the entire 1 year during follow-up. There was no significant compression and the reason of headache was still unknown. Of the 68 patients with ACTH adenomas, 61(89.7%) showed tumor volume decrease or remain unchanged and 19(27.9%) experienced normalization of hormone level (Figure 1 and Figure 2). Of the other 5 patients with enlarged ACTH adenomas, 4 had repeated MASEP GKRS. One had craniotomy and resection of the mass after experiencing consistent vomiting. Another two cases with no clinical symptom with a neuroradiological diagnosis of radiation necrosis received no more treatment. Of the 176 patients with prolactinomas, 41(23.3%) had normalization of hormone level and 159(90.3%) showed tumor volume decrease or remain unchanged (Figure 3 and Figure 4). Of the 12 patients with enlarged prolactinomas, 9 had repeated GKRS. Two had transsphenoidal resection of the mass after experiencing consistent headache. One case died 4 years after primary MASEP GKRS rejecting any medical intervention. Another 5 cases with the diagnosis of radiation necrosis had no clinical symptoms and lived as usual. Of the 103 patients with GH adenomas, 98(95.1%) experienced tumor volume decrease or remain unchanged and 38(36.9%) showed normalization of hormone level (Figure 5 and Figure 6). Of the other 3 patients with enlarged GH adenomas, 2 had repeated MASEP GKRS. One had craniotomy and resection of the mass after experiencing consistent vomiting. Another 2 cases with no clinical treatment had a neuroradiological diagnosis of radiation necrosis and were under observation.

Regular endocrinological and neuroradiological re-examinations were available in all these patients. The data collected as of the end of 2007 are summed up in table 3 and table 4.

**Table 3:** Neuroradiological changes of patients with pituitary adenomas treated with MASEP GKRS

Type of adenomas	collapse	unchanged	enlarge	enlarged with necrosis
ACTH adenomas				
microadenoma	5	14	2	0
macroadenoma	23	19	3	2
Prolactinomas				
microadenoma	0	0	0	0
macroadenoma	97	62	12	5
GH adenomas				
microadenoma	0	0	0	0
macroadenoma	56	42	3	2

Total(%)	181(52.1)	137(39.5)	20(5.8)	9(2.6)

4 patients with ACTH adenomas had repeated MASEP GKRS; 12 patients with prolactinomas had repeated MASEP GKRS; 2 patients with GH adenoma had repeated MASEP GKRS

**Table 4:** Endocrinological changes of patients with pituitary adenomas treated with MASEP GKRS

Type of adenomas	normalization	decrease	no improve	hypopituitarism
ACTH adenomas				
microadenoma	7	11	2	1
macroadenoma	12	31	4	0
Prolactinomas				
microadenoma	0	0	0	0
macroadenoma	41	114	18	3
GH adenomas				
microadenoma	0	0	0	0
macroadenoma	38	56	7	2

Total(%)	98(28.2)	212(61.1)	31(8.9)	6(1.7)

Hypopituitarism occurred in 1 patients with ACTH adenomas after MASEP GKRS; 3 patients with prolactinomas had hypopituitarism after MASEP GKRS; 2 patient with GH adenoma had hypopituitarism after MASEP GKRS

Overall 91.6% of tumor control was achieved in 318 with only mild and transient neurological complications in some cases. 28.2% of normalization of hormone level rate and 61.1% of decrease of hormone level rate were also achieved. Hypopituitarism occurred in 6(1.7%) patients who received replacement therapy now.

## Discussion

There are multiple treatment modalities for pituitary adenomas. The individual treatment must be tailored to a patient's symptoms, overall health, and tumor morphometry. GKRS has been found to be an effective, noninvasive method for treating patients with functioning pituitary adenoma as a complement to the surgery. Tumors that compress the optic pathway should be removed with microsurgery, and residual tumor, especially in the cavernous sinus, is a good indication for radiosurgery. At our institute, MASEP GKRS may be considered as an alternative treatment to microsurgery if patients are reluctant to undergo surgical resection, or are unable to undergo microsurgery under general anesthesia because of old age or poor medical conditions. The purpose of GKRS, in the case of secretory pituitary adenomas, is to control tumor growth and normalize endocrinological hypersecretion. Secretory adenomas seem to require a higher radiation dose than nonfunctioning pituitary adenomas[13]. Ganz suggested that the effective dose for secretory adenomas should be higher than 25 Gy according to the details[14]. Laws and Vance estimated that a higher percentage of control of hyper-functioning syndromes could be accomplished with the higher margin dose[15]. The lowest effective radiation dose in our study was 12 Gy delivered to the tumor margin; the mean marginal dose was 22.2 Gy. According to our experience, the suitable margin dose should depend on the endocrinological type of the secretory pituitary adenoma. However, the recent report of Pollock for functioning adenomas revealed the radiation dose was not related to endocrinological outcome[16].

In nearly all published series, stereotactic radiosurgery afforded excellent control of tumor growth. Hayashi reported that the tumor control rate for pituitary adenoma after GKRS was between 93% and 94%, and that the tumor shrinkage rate ranged from 46% to 56.7%[17]. Many studies reported a greater than 95% control of tumor size with follow-up varying from months to years[18,19]. Some series have even demonstrated improvement in visual function following radiosurgery upon shrinkage of the tumor. Most pituitary adenomas tend to be slow growing lesions. As such, it may be misleading to evaluate series of patients with relatively short follow-up. In our previous study, the effects of MASEP GKRS may get stable within three years after the treatment, and this study shows concordant results within the follow-up more than 5 years.

At the time when GKRS started, the results of microsurgery were disappointing regarding ACTH-producing pituitary adenomas and the role of GKRS as primary therapy was evaluated. We have not seen any recurrences after MASEP GKRS in patients who obtained remission in contrast to pituitary microsurgery with progressive increase of recurrences of Cushing's syndrome with time. Cushing's disease is a serious catabolic illness that requires rapid normalization of cortisol hypersecretion. Thus pituitary microsurgery is the primary treatment for Cushing's disease; gamma knife surgery can be applied when open surgery is contraindicated or refused or as a secondary treatment when open surgery has failed or the tumor extends into the cavernous sinuses. Many series utilized the 24 h urine cortisol collection as part of the criteria for endocrinological evaluation, and the endocrinological'cure'rates ranged from 17 to 83%[20,21]. In a recent review, Laws and Vance reported remission in about 60% of their patients with Cushing's disease followed for more than 6 months with a mean time to remission of approximately one year when gamma knife surgery was used as a complement to open surgery[22]. In this research, we observed similar result in our patients with radiosurgery as the major treatment.

As most patients with prolactinomas can be adequately controlled by medical treatment. Gamma knife radiosurgery has been used by us in only few patients. It may be a suitable alternative in patients who experience side effects of dopaminergic drugs or in patients with tumor extension to the cavernous sinuses. The largest series of prolactinomas treated with GKRS was reported by Pan et al[23]. Their study used normal serum prolactin level for gender as cure criteria, and they reported a 15% endocrinological remission rate achieved for 128 patients with a median follow-up of 33 months. Some studies utilize relatively similar criteria. 'Cure'rates varied from 20 to 84%. In our study, we achieved better tumor growth control than endocrinological control without the use of medical therapies after radiosurgery, and the usage of medical therapies after radiosurgery still needed further evaluation. Pan et al suggested that dopaminergic drugs seemed to induce radioprotection[23]. In our unit, MASEP GKRS were performed during an intermission in drug therapy when the drug therapy is discontinued.

The criteria for controlling acromegaly have still been inconsistent. The most widely accepted guidelines for a remission in acromegaly consist of a GH level less than 1 ng/ml in response to a glucose challenge and a normal serum IGF-1 when matched for age and gender. Some studies with such criteria detail the results of GKRS for patients with acromegaly. The mean radiosurgery margin doses in these series ranged from 15 to 34 Gy. 'Cure'rates following radiosurgery varied from 0 to 100%. In these series with at least 16 patients and a median follow-up of 2 years, endocrinological remission rates ranged from 20 to 96%[24,25]. Our study found similar results with longer follow-up. The high incidence of hypopituitarism is one of the significant shortcomings of conventional radiotherapy[26]. It can develop many years after irradiation. The data available are varied, depending on the length of follow-up. Tsang reported more than 22% of patients developing hypopituitarism during the 10 years after conventional irradiation[27]. Salinger reported 37% of patients developing hypopituitarism, within a follow-up of 5 years[28]. Stereotactic targeting, allowed by GKRS, should lower the incidence of hypopituitarism. However, the incidence of hypopituitarism after GKRS is difficult to determine at present. Reports in the literature for the incidence of post-radiosurgery hypopituitarism vary widely. Well respected groups have reported a low incidence (0~36%) of pituitary dysfunction following radiosurgery[29]. A long term study from the Karolinska Institute with a mean follow-up of 7 years, however, reported an eventual 42% incidence of hypopituitarism[30]. Post found that hypopituitarism following radiosurgery correlated with the radiation dose to the pituitary stalk[31]. The difficulty with determining the exact incidence of radiosurgery-induced hypopituitarism stems in part from the fact that many of the patients have already undergone previous radiation therapy or surgery. In addition, pituitary deficiencies may result in part from normal aging. Thus, it is likely that hypopituitarism in the post-radiosurgical population is multifactorial in etiology and related to radiosurgery as well as to age-related changes and previous treatments. However, in 347 patients with secretory pituitary adenomas treated, only 1.7% patients developed hypopituitarism. The MASEP rotary gamma knife may make an important contribution to this result. The 25 60-Co sources were all rotating during the whole treatment process and the healthy pituitary stalk received much less dose of irradiation than in the radiosurgery with traditional static gamma knife. We proposed that the dose of irradiation on pituitary tissue may be the most important cause of hypopituitarism. Kokubo reported the similar findings[32].

## Conclusion

In summary, MASEP GKRS can be an effective method for controlling tumor growth and inducing hormonal normalization in patients with functioning pituitary. The treatment is safe with low mortality and morbidity. Complications from the optic apparatus have not been found when the dose to that area is below 10 Gy. Brain necrosis, neuropsychological disturbances and secondary brain tumors have not been found with gamma knife radiosurgery. The incidence of post-radiosurgery hypopituitarism is very low and the development of hypopituitarism following radiosurgery can be avoided by observing the maximum mean dose on healthy peritumoral pituitary of 15 Gy according to our experience. In our treatment, the rotary gamma knife is proved to be as safety and efficient as the static gamma knife. Long-term follow up after MASEP GKRS for control of pituitary function is still needed even when the patient is in remission due to the risk of late occurring pituitary insufficiency.

## Competing interests

The authors declare that they have no competing interests.

## Authors' contributions

HW carried out the follow-up of the patients, participated in the irradiation treatment and drafted the manuscript. OC established this gamma knife centre and participated in the irradiation treatment. SBY conceived of the study, and participated in its design and coordination and helped to draft the manuscript. All authors read and approved the final manuscript.
